# Recombinant human erythropoietin alters gene expression and stimulates proliferation of MCF-7 breast cancer cells

**DOI:** 10.2478/raon-2013-0056

**Published:** 2013-10-08

**Authors:** Nina Trost, Tina Stepisnik, Sabina Berne, Anja Pucer, Toni Petan, Radovan Komel, Natasa Debeljak

**Affiliations:** 1 Center for Functional Genomics and Bio-chips, Institute of Biochemistry, Faculty of Medicine, University of Ljubljana, Slovenia; 2 Medical Center for Molecular Biology, Institute of Biochemistry, Faculty of Medicine, University of Ljubljana, Slovenia; 3 Department of Molecular and Biomedical Sciences, Jožef Stefan Institute, Ljubljana, Slovenia

**Keywords:** breast cancer, erythropoietin, erythropoietin receptor isoforms, proliferation, gene expression

## Abstract

**Background:**

Functional erythropoietin (EPO) signaling is not specific only to erythroid lineages and has been confirmed in several solid tumors, including breast. Three different isoforms of erythropoietin receptor (EPOR) have been reported, the soluble (EPOR-S) and truncated (EPOR-T) forms acting antagonistically to the functional EPOR. In this study, we investigated the effect of human recombinant erythropoietin (rHuEPO) on cell proliferation, early gene response and the expression of *EPOR* isoforms in the MCF-7 breast cancer cell line.

**Materials and methods:**

The MCF-7 cells were cultured with or without rHuEPO for 72 h or 10 weeks and assessed for their growth characteristics, expression of early response genes and different *EPOR* isoforms. The expression profile of *EPOR* and *EPOR-T* was determined in a range of breast cancer cell lines and compared with their invasive properties.

**Results:**

MCF-7 cell proliferation after rHuEPO treatment was dependent on the time of treatment and the concentration used. High rHuEPO concentrations (40 U/ml) stimulated cell proliferation independently of a preceding long-term exposure of MCF-7 cells to rHuEPO, while lower concentrations increased MCF-7 proliferation only after 10 weeks of treatment. Gene expression analysis showed activation of *EGR1* and *FOS*, confirming the functionality of EPOR. rHuEPO treatment also slightly increased the expression of the functional *EPOR* isoform, which, however, persisted throughout the 10 weeks of treatment. The expression levels of *EPOR-T* were not influenced. There were no correlations between *EPOR* expression and the invasiveness of MCF-7, MDA-MB-231, Hs578T, Hs578Bst, SKBR3, T-47D and MCF-10A cell lines.

**Conclusions:**

rHuEPO modulates MCF-7 cell proliferation in time- and concentration-dependent manner. We confirmed *EGR1*, *FOS* and *EPOR* as transcription targets of the EPO-EPOR signaling loop, but could not correlate the expression of different *EPOR* isoforms with the invasiveness of breast cancer cell lines.

## Introduction

Erythropoietin (EPO) is a 34 kDa glycoprotein hormone that regulates erythroid maturation in bone marrow.^1^ Its binding to the erythropoietin receptor (EPOR) on the surface of erythroid progenitors triggers several downstream signaling pathways, including Janus kinase 2 (Jak2)/signal transducer and activator of transcription 5 (STAT5), phosphatidylinositol 3-kinase (PI3K)/protein kinase B (Akt), Ras/mitogen-activated protein kinase (MAPK) and protein kinase C (PKC) pathways.^2^ EPO-EPOR signaling not only promotes erythroid proliferation and differentiation, but also protects erythroid progenitors against apoptosis.^3^ EPO has been shown to increase the frequency of S-phase burst-forming-units (BFUs) in human bone marrow.^4^ Furthermore, EPO increases the expression of anti-apoptotic proteins B-cell lymphoma 2 (Bcl-2) and B-cell lymphoma-extra large (Bcl-X_L_) via the Jak2/STAT5 signaling pathway.^5^ Functional EPO-EPOR signaling is not limited only to erythroid lineages since EPOR expression has been confirmed in several non-hematopoietic cells and tissues, as well as in solid tumors.^6^ Recombinant forms of human erythropoietin (rHuEPO), used in clinical oncology settings to improve anemia, have been correlated with lower survival rates of patients undergoing rHuEPO treatment.^2^ These observations raised concerns about EPO’s potential in promoting cancer growth and development of more aggressive cancer phenotypes. Therefore, EPO-EPOR signaling has been studied in correlation to cancer progression in several laboratories. Their findings are conflicting and strongly depend on the used experimental models, as rHuEPO was reported to increase cancer cell proliferation^7^,^8^ or to have no significant effect.^9^,^10^ Contrasting effects might be explained by the presence of different EPOR isoforms. Three EPOR isoforms are listed in the UniProt database (http://www.uniprot.org/uniprot/P19235): a full-length functional (EPOR-F), a truncated isoform (EPOR-T) lacking the cytoplasmic region 11 and a soluble (EPOR-S) receptor that is missing the trans-membrane and cytoplasmic domains.^12^ EPOR-S is secreted from the cell where it competes with EPOR-F for EPO binding.^13^ The EPOR-T and EPOR-S isoforms most probably act as antagonists of EPOR-mediated signaling.^14^ All three isoforms were confirmed in breast cancer.^15^

The objective of our study was to investigate the effect of rHuEPO on cell proliferation, *EPOR* expression and early gene response in breast cancer cells. The effect of a long-term rHuEPO treatment of MCF-7 cells on cell proliferation, EPO-responsiveness and the expression of functional (*EPOR*), soluble (*EPOR-S*) and truncated (*EPOR-T*) receptor isoforms was assessed. Additionally, the expression profile of *EPOR* and *EPOR-T* was determined in a range of breast cancer cell lines and compared with their invasive properties.

## Materials and methods

### Cell lines

The breast cancer cell lines (Table 1) were from the American Type Culture Collection (ATCC; Manassas, VA, USA) and were cultured according to their recommendations in basic growth medium, supplemented with 10% fetal bovine serum (FBS) at 37˚C in a humidified 5% (v/v) CO_2_ atmosphere. The receptor status of a specific cell line and the tumor type are shown in Table 1. The MCF-7 cells were pretreated with rHuEPO up to 10 weeks (5 U/ml, NeoRecormon, Roche, Germany). In parallel, control cells were cultured in the same conditions, but without rHuEPO.

### Proliferation assays

The effect of rHuEPO on cell proliferation was analyzed using the colorimetric 3-(4,5-dimethylthiazol-2-yl)-2,5-diphenyltetrazolium bromide (MTT, Sigma, USA) assay. rHuEPO pretreated (10 weeks) and non-pretreated cells were seeded in a volume of 100 μl on a 96-well plate at a density of 5 × 10^3^ cells per well. Cells seeded in six replicates were left to adhere for 24 h. Growth medium was then replaced with a medium supplemented with different concentrations of rHuEPO (0, 5, 40 U/ml). Cells were grown for 72 h and at specific time-points 15 μl of MTT (5 mg/ml in PBS) was added to each well and the plate was incubated at 37°C for 3 h, according to the manufacturer’s recommendations. Cell metabolic activity reflecting cell number and thus proliferation was measured daily and normalized to values obtained with control cells not exposed to rHuEPO.

### Gene expression analysis

#### Sample preparation

**FIGURE 1.**
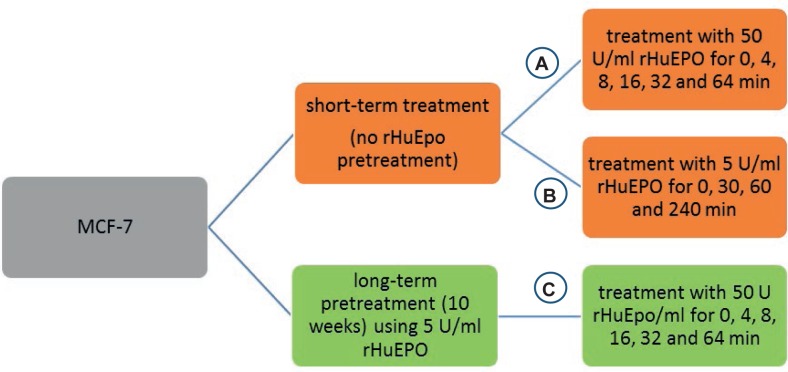
Protocol of treatment of MCF-7 cells with recombinant human erythropoietin for isolation of total RNA.

MCF-7 cells pretreated with rHuEPO for 10 weeks (Figure 1C) and non-pre-treated cells (Figure 1A) were cultured in basic growth medium in T-25 flasks at a density of 5 × 10^5^ cells/ml and grown to 75% of confluency. Cells were serum starved for 24 h and exposed to 50 U/ml rHuEPO for 0, 4, 8, 16, 32 and 64 min. Following the stimulation with rHuEPO, cells were subjected to RNA isolation and analyzed for *EPOR* expression levels. The non-pretreated cells were further analyzed for early gene response. Cells were cultured in 6-well plates at a density of 3 × 10^5^ cells/ml in serum-deprived media and cultured for 48 h. Cells were stimulated with 5 U/ml rHuEPO for 0, 30, 60 and 240 min, fast frozen in liquid nitrogen and subjected to RNA isolation (Figure 1B).

#### RNA isolation

Total RNA was isolated using the High Pure Total RNA Isolation Kit (Roche) or TRI Reagent (Sigma) following manufacturer’s instructions. The Agilent Bioanalyzer 2100 (Agilent Technologies, USA) was used for the determination of RNA concentrations and quality, assuring all RNA integrity numbers (RINs) were above 9.8. Total RNA was transcribed to cDNA using Transcriptor First Strand cDNA Synthesis Kit (Roche) and SuperScript III reverse transcriptase (Invitrogen, USA).

#### Quantitative real-time PCR (qPCR)

Forward and reverse primers for *FOS, JUN, NFκ*B*, FOSL1, EGR1, RPLP0* and *GAPDH* were designed to span intron-exon junctions using PrimerExpress software (Applied Biosystems, USA) and their specificity was checked using BLAST algorithm (Table 2). *RPLP0* and *GAPDH* were used as reference genes in the analysis of early gene response. Forward and reverse primers for functional (*EPOR*), soluble (*EPOR-S*) and truncated (*EPOR-T*) erythropoietin receptor were designed according to Arcasoy *et al.*^15^ Primers specific for *SF3A1* and *YWHAZ* genes from the Human geNorm Kit (Primer Design, UK) and for *TOP1*^17^ were chosen as reference genes in the analysis of the *EPOR* isoform expression. Primer validation was done by analyzing the slope of the standard curve and the presence of a single peak in the melting curve after qPCR analysis. qPCR was conducted on a 384-well plates using the LightCycler 480 Real-Time PCR System (Roche) and SYBR Green I Master chemistry (Roche). Amplification of specific PCR products was performed in triplicates in a total reaction mixture of 5 μl, containing 750 ng RNA equivalent cDNA template and 300 nM of each set of primers. The expression levels of the selected reference genes were used for normalization of expression data. Gene expression normalization factors were calculated for each sample based on geometric means of the selected reference genes.^18^ Minimum Information for Publication of Quantitative Real-Time PCR Experiments (MIQE) was followed in the performance and interpretation of the qPCR reactions.^19^

### *EPOR* expression and cancer invasiveness

The invasiveness of breast cancer cell lines was compared with the expression of *EPOR* isoforms. Cell lines differing in cell invasiveness as represented in Table 1.

### Statistical analysis

Statistical analysis of the data was performed using the Limma package^20^ from Bioconductor analysis tools for R programming language.^21^ The effect of rHuEPO treatment on cell proliferation and gene expression was assessed by Two-way analysis of variance (ANOVA). Multiple-testing correction using False discovery rate (FDR)^22^ was employed and p < 0.05 was considered as statistically significant.

## Results

### EPO alters the proliferation rate of MCF-7 breast cancer cells

**FIGURE 2.**
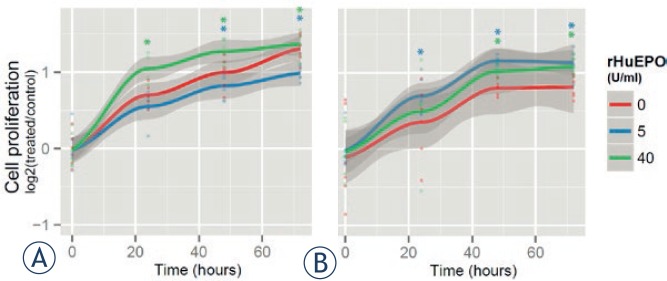
Differential effects of recombinant human EPO on MCF-7 cell proliferation (A) MCF-7 cells were cultured in complete medium in the presence of indicated concentrations of rHuEPO (short-term treated) (B) MCF-7 cells were cultured in complete medium in the presence of 5 U/ml of rHuEPO for 10 weeks (long-term pretreated cells), EPO was added to the pretreated cells at indicated concentrations. Asterisk (*) denotes statistical significance for Type 1 error α = 0.05.

MCF-7 cells were stimulated with rHuEPO (0, 5, 40 U/ml) and assessed for proliferation using the MTT assay. We found that MCF-7 cell proliferation is dependent on the concentration of rHuEPO used and the time of the treatment (Figure 2). Treatments with 40 U/ml rHuEPO led to increased MCF-7 cell proliferation independently of the length of cell exposure to rHuEPO. On the other hand, 5 U/ml rHuEPO affects MCF-7 cell proliferation in a time dependent manner; cell proliferation was reduced during a short-term treatment (Figure 2A), but was higher when rHuEPO was added to long-term rHuEPO-pretreated cells (Figure 2B).

### EPO induces gene expression changes in MCF-7 cells

#### The expression of EPOR isoforms in EPO-treated cells

**FIGURE 3.**
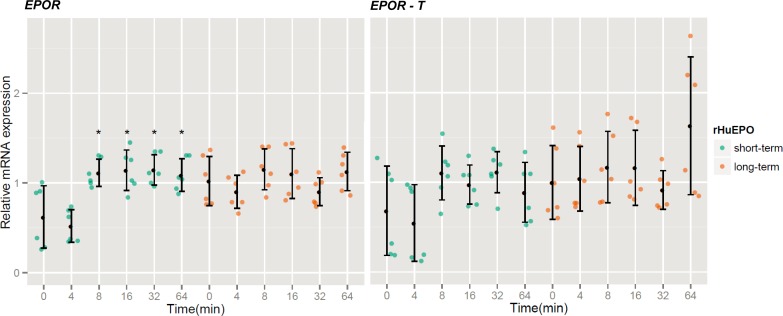
Effects of recombinant human EPO on relative *EPOR* and *EPOR-T* expression. MCF-7 cells were stimulated with 50 U/ml rHuEPO (short-term, green) or cultured in complete medium in the presence of 5 U/ml of rHuEPO for 10 weeks and stimulated with 50 U/ml rHuEPO (long-term, red). Error bars represent standard deviations (SD) between six replicate samples; asterisk (*) denotes statistical significance for Type 1 error α = 0.05.

To determine the effects of rHuEPO on the expression of its receptor protein variants, mRNA expression levels of *EPOR*, *EPOR-S* and *EPOR-T* genes were analyzed in short (Figure 1A) and long-term (Figure 1C) rHuEPO-treated MCF-7 cells. The expression of *EPOR* and *EPOR-T* isoforms at specific time-points was confirmed by qPCR (Figure 3). On the other hand, we were not able to confirm the presence of *EPOR-S* (data not show). Short-term stimulation of MCF-7 cells with 50 U/ml rHuEPO leads to an increase in *EPOR* expression, while it has no statistically significant effect on *EPOR-T*. Interestingly, the addition of 50 U/ml rHuEPO to the long-term pretreated cells (5 U/ml rHuEPO) did not have any additional influence on the expression levels of *EPOR* and *EPOR-T*.

#### The expression of early response genes in EPO-treated cells

**FIGURE 4.**
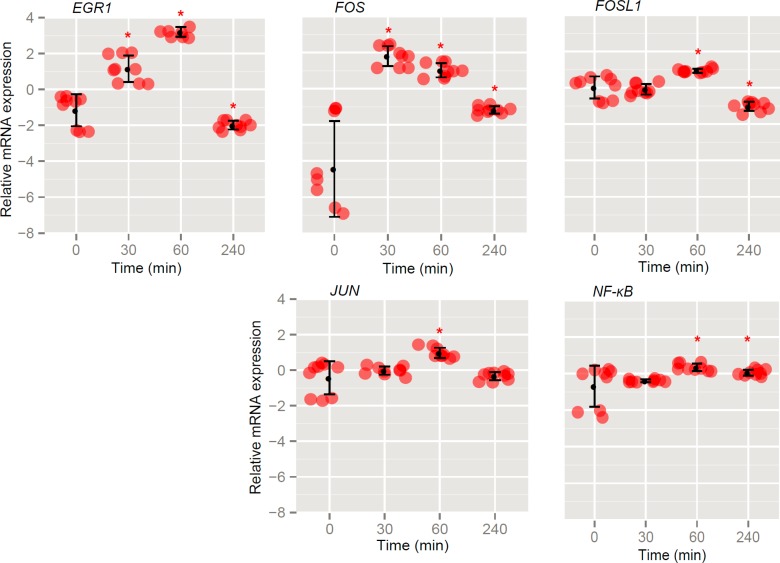
Early gene response upon rHuEPO stimulation of MCF-7 cells. The expression of *EGR1, FOS, FOSL1, JUN* and *NF-κB* was determined at the indicated time-points during rHuEPO treatment (5 U/ml) of MCF-7 cells grown in serum-stripped growth medium. Error bars represent standard deviations (SD) determined from six replicate samples; asterisk (*) denotes statistical significance for Type 1 error α = 0.05.

Since rHuEPO affected MCF-7 cell proliferation in a time-dependent manner only at the 5 U/ml concentration, MCF-7 cells were stimulated with 5 U/ml rHuEPO and analyzed for early gene response. The most pronounced changes were observed in the expression of *EGR1* and *FOS* (Figure 4). Both genes were up-regulated after rHuEPO stimulation. rHuEPO only slightly modulated the expression of *FOSL1, JUN* and *NF-κB* genes.

### The expression of *EPOR* does not correlate with breast cancer cell invasiveness

**FIGURE 5.**
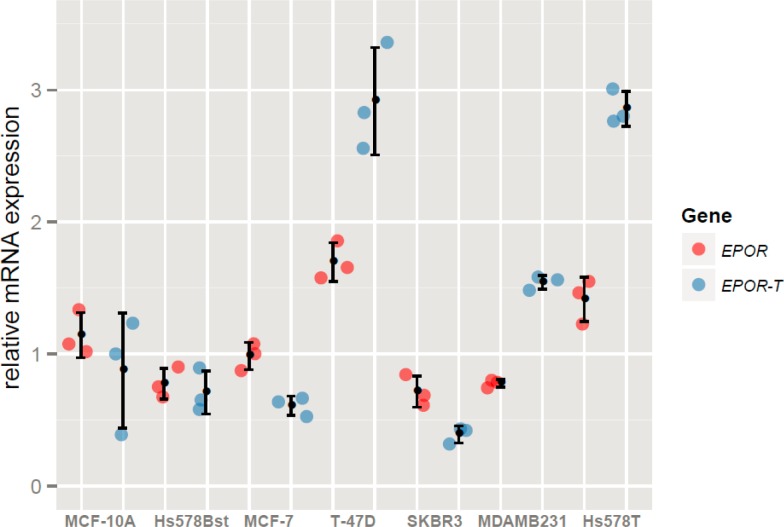
Expression of *EPOR* isoforms in different breast cancer cell lines; expression of functional *EPOR* (red); expression of truncated form of *EPOR-T* (blue). Cell lines differ in the level of invasiveness with MCF-10A cell line being the least invasive and Hs578T cell line being the most invasive (Table 1). Error bars represent standard deviations (SD) of the relative expression values determined in triplicate samples.

The expression of *EPOR* isoforms was paralleled with the invasiveness of cancer and epithelial-like breast cell lines included in the current study (Table 1). We found no association between the expression of *EPOR* (*EPOR* or *EPOR-T*) and the breast cell invasiveness. There were no significant differences in the level of *EPOR* expression between cell lines and its expression in a particular cell line did not correlate with its invasiveness, ESR, PGR or HER2 status (Figure 5).

## Discussion

EPO is a key regulator of erythropoiesis and is gaining more significance also in other tissues^2^,^6^ and (patho)physiological processes. EPO is important for neuro-23 and cardioprotection24, while the functionality of EPO-EPOR signaling in cancer settings questions the suitability of its usage for the treatment of cancer or chemotherapy-related anemia.^25^ EPOR activation is considered to influence cancer cell growth in terms of stimulated proliferation, prevention of apoptosis and increased resistance to therapy. The mechanisms of EPO actions are not well understood, but it has been suggested that an active crosstalk with other growth factor receptors is involved, especially those from the estrogen family and HER2.^26^,^27^ It has been shown that the AP-1 (FOS and JUN) transcription factor is critical for growth and proliferation of breast cancer cells.^28^ We therefore analyzed early gene response in MCF-7 cells stimulated with rHuEPO. We show that rHuEPO induces rapid up-regulation of *FOS* and *EGR1* gene expression, which is followed by an increase in the expression of *JUN* and *NF-κB* (Figure 4). Despite the up-regulation of *FOS*29 and *EGR1*^30^ genes, both considered a driving force for cell proliferation, we observed a decreased proliferation rate of short-term (72 h) treated MCF-7 cells after stimulation with rHuEPO (5 U/ml) (Figure 2). On the contrary, the effect was reversed after long-term pretreatment being in agreement with our previously published data.^31^ This suggests that a long-term treatment with low doses of rHuEPO sensitizes the MCF-7 cells to further treatment with the growth factor. At a higher concentration of 40 U/ml, rHuEPO significantly increased cell proliferation independently of the any previous exposure of MCF-7 cells to the hormone.

Further, we analyzed the expression of functional *EPOR* and its antagonists, truncated (*EPOR-T*) and soluble *EPOR* (*EPOR-S*), in rHuEPO-treated MCF-7 cells and other breast cancer cell lines. The presence of *EPOR-S* was not confirmed, despite previous reports of its presence in MCF-7 cells.^32^ We found no association between the expression of *EPOR* (*EPOR or EPOR-T*) and the breast cell invasiveness. There were no significant differences in the level of EPOR expression between cell lines and its expression in a particular cell line did not correlate with its invasiveness, ESR, PGR or HER2 status (Figure 5).

Interestingly, we show here that rHuEPO can slightly up-regulate the expression of the functional *EPOR,* but has no effect on *EPOR-T*. The up-regulation of functional *EPOR* is very fast, it happens after 8 min of rHuEPO (50 U/ml) stimulation. The addition of 50 U/ml rHuEPO to the long-term pretreated cells (5 U/ml rHuEPO) did not have any additional influence on the *EPOR* expression levels. It seems the expression is slightly elevated throughout whole long-term treatment (Figure 3). Our results indicate that rHuEPO stimulation regulates the expression of *EPOR* but not *EPOR-T* in MCF-7 cells as indicated previously.^33^ Finally, the analysis of *EPOR* mRNA levels in a panel of breast cancer cell lines suggests that the pattern of *EPOR* (functional and *EPOR-T*) expression does not correlate with the invasiveness of breast cancer cell lines (Figure 5).

## Conclusions

Our study confirmed the functionality of EPOEPOR signaling pathways in MCF-7 cells, indicating time- and concentration-dependent rHuEPO effects on cell proliferation. The 5 U/ml (physiological) rHuEPO concentration was shown to have an opposite effect on cell proliferation after 10 weeks versus 72 hours of treatment, most probably due to cell line sensibilization. Furthermore, two *EPOR* isoforms were confirmed, full-length functional *EPOR* and truncated *EPOR-T*, showing different expression profile upon rHuEPO treatment. The observed expression profiles are not correlated with the invasiveness of analyzed breast cancer cell lines.

## Figures and Tables

**TABLE 1.** Details on the cohort of breast cancer cell lines as defined by ATCC. ESR, estrogen receptor; PGR, progesterone receptor; AC, adenocarcinoma; IDC, invasive ductal carcinoma; F, fibrocystic disease; PE, pleural effusion; P. Br, primary breast. Cell invasiveness increases with number (1 = the least invasive, 7 = the most invasive). Cells were cultured as described in Hevir *et al.*^16^

**Cell line**	**Receptor status**	**Tissue source**	**Tumor type**	**Invasiveness**
**MCF-10A**	ESR–, PGR–		F	1
**Hs578Bst**	ESR–, PGR–	Adjacent breast tissue		2
**MCF-7**	ESR+, PGR+	PE	IDC	3
**T-47D**	ESR+, PGR+	PE	IDC	4
**SK-BR-3**	ESR–, PGR–, HER2+	PE	AC	5
**MDA-MB-231**	ESR–, PGR–	PE	AC	6
**Hs578T**	ESR–, PGR–	P. Br	IDC	7

**TABLE 2.** Primers used in qPCR analysis of genes of interest and reference genes. Forward (Fw) and reverse (Rev) reverse oligonucleotide primers are shown; (NA) not available

**Genes of interest**					
**Gene symbol**	**Gene name**	**Nucleotide sequence**	**Ref. seq.**	**Amplicon length**	**PCR Eff**
**EPOR**		*Fw: 5′-GCTGGAAGTTACCCTTGTGG-3′* *Rev: 5′-CTCATCCTCGTGGTCATCCT-3′*	NM_000121	148	1.920
**EPOR-T**	erythropoietin receptor, truncated form	*Fw: 5′-GGTCCAGGTCGCTAGGCGTCAG-3′* *Rev: 5′-TGCTTCTTGCAGCCAAACTGC-3′*	NM_000121	249	1.911
**EPOR-S**	erythropoietin receptor, soluble form	*Fw: 5′-CTCCACCCTCTGTACGCTCCCTGC-3′* *Rev: 5′-ACGCCTAGCGGGCTCTGAAGC-3′*	NM_000121	183	(NA)
**FOS**	FBJ murine osteosarcoma viral oncogene homolog	Fw: 5′-CTACCACTCACCCGCAGACT-3′ Rev: 5′-AGGTCCGTGCAGAAGTCCT-3′	NM_005252.2	72	2
**JUN**	jun-proto oncogene	Fw: 5′-CCAAAGGATAGTGCGATGTTT-3′ Rev: 5′-CTGTCCCTCTCCACTGCAAC-3′	NM_002228.2	62	2
**NF-κB**	nuclear factor of kappa light polypeptide gene enhancer in B-cells 1	Fw: 5′-GGTGCCTCTAGTGAAAAGAACAAGA-3′ Rev: 5′-GCTGGTCCCACATAGTTGCA-3′	NM_003998.3	68	1.722
**FOSL1**	FOS-like antigen 1	Fw: 5′-AACCGGAGGAAGGAACTGAC-3′ Rev: 5′CTGCAGCCCAGATTTCTCAT-3′	NM_005438.3	75	2
**EGR1**	Early growth response 1	*Fw: 5′-AGCCCTACGAGCACCTGAC-3′;* *Rev: 5′-GGTGGTGGGGTAACTG-3′*	NM_001964.2	81	2

**Reference genes**					
**Gene symbol**	**Gene name**	**Nucleotide sequence**	**Ref. seq.**	**Amplicon length**	**Primer Eff**
**RPLP0**	ribosomal protein, large, P0	Fw: 5′-TCTACAACCCTGAAGTGCTTGAT-3′ Rev: 5′-CAATCTGCAGACAGACACTGG-3′	NM_001002.3	96	2.073
**GAPDH**	glyceraldehyde-3-phosphate dehydrogenase	Fw: 5′-AGCCACATCGCTCAGACAC-3′ Rev: 5′-GCCCAATACGACCAAATCC-3′	NM_002046.3	66	1.999
**SF3A1**	Splicing factor 3a, subunit 1	NA	NM_005877	NA	1.799
**TOP1**	DNA Topoisomerase I	Fw: 5′-CCCTGTACTTCATCGACAAGC-3′ Rev: 5′-CCACAGTGTCCGCTGTTTC-3′	NM_003286.2	NA	1.809
**YWHAZ**	Tyrosine 3-monooxygenase/tryptophan 5-monooxygenase activation protein, zeta polypeptide	NA	NM_003406	NA	1.887
